# Uncovering the Bronchoalveolar Single-Cell Landscape of Patients With Pulmonary Tuberculosis With Human Immunodeficiency Virus Type 1 Coinfection

**DOI:** 10.1093/infdis/jiae042

**Published:** 2024-02-27

**Authors:** Guohui Xiao, Waidong Huang, Yu Zhong, Min Ou, Taosheng Ye, Zhifeng Wang, Xuanxuan Zou, Feng Ding, Yuan Yang, Zhe Zhang, Chuanyu Liu, Aimei Liu, Longqi Liu, Shuihua Lu, Liang Wu, Guoliang Zhang

**Affiliations:** National Clinical Research Center for Infectious Diseases, Shenzhen Third People's Hospital, Southern University of Science and Technology, Shenzhen; BGI Research, Shenzhen; College of Life Sciences, University of Chinese Academy of Sciences, Beijing; BGI Research, Shenzhen; National Clinical Research Center for Infectious Diseases, Shenzhen Third People's Hospital, Southern University of Science and Technology, Shenzhen; National Clinical Research Center for Infectious Diseases, Shenzhen Third People's Hospital, Southern University of Science and Technology, Shenzhen; BGI Research, Shenzhen; BGI Research, Shenzhen; College of Life Sciences, University of Chinese Academy of Sciences, Beijing; National Clinical Research Center for Infectious Diseases, Shenzhen Third People's Hospital, Southern University of Science and Technology, Shenzhen; BGI Research, Shenzhen; MGI Tech, Shenzhen; BGI Research, Shenzhen; BGI Research, Hangzhou; Department of Tuberculosis, Guangxi Chest Hospital, Liuzhou; BGI Research, Shenzhen; BGI Research, Hangzhou; National Clinical Research Center for Infectious Diseases, Shenzhen Third People's Hospital, Southern University of Science and Technology, Shenzhen; BGI Research, Shenzhen; BGI Research, Chongqing, China; National Clinical Research Center for Infectious Diseases, Shenzhen Third People's Hospital, Southern University of Science and Technology, Shenzhen

**Keywords:** single-cell RNA sequencing, pulmonary TB, HIV-1, bronchoalveolar lavage fluid, local immunity

## Abstract

**Background:**

Coinfection of human immunodeficiency virus type 1 (HIV-1) is the most significant risk factor for tuberculosis (TB). The immune responses of the lung are essential to restrict the growth of *Mycobacterium tuberculosis* and avoid the emergence of the disease. Nevertheless, there is still limited knowledge about the local immune response in people with HIV-1–TB coinfection.

**Methods:**

We employed single-cell RNA sequencing (scRNA-seq) on bronchoalveolar lavage fluid from 9 individuals with HIV-1–TB coinfection and 10 with pulmonary TB.

**Results:**

A total of 19 058 cells were grouped into 4 major cell types: myeloid cells, T/natural killer (NK) cells, B cells, and epithelial cells. The myeloid cells and T/NK cells were further divided into 10 and 11 subsets, respectively. The proportions of dendritic cell subsets, CD4^+^ T cells, and NK cells were lower in the HIV-1–TB coinfection group compared to the TB group, while the frequency of CD8^+^ T cells was higher. Additionally, we identified numerous differentially expressed genes between the CD4^+^ and CD8^+^ T-cell subsets between the 2 groups.

**Conclusions:**

HIV-1 infection not only affects the abundance of immune cells in the lungs but also alters their functions in patients with pulmonary TB.

## Teaser

We utilized single-cell RNA sequencing technology to provide a detailed analysis of immune cells in the lungs of patients with tuberculosis (TB) or with HIV–TB coinfection. Our findings revealed notable variations in immune response between these 2 disease types.

Individuals with human immunodeficiency virus (HIV) are more susceptible to developing active tuberculosis (TB) from either a new exposure to *Mycobacterium tuberculosis* (*Mtb*) or a latent *Mtb* infection than those who are HIV negative [1]. It is well known that CD4^+^ T cells are the primary target of HIV type 1 (HIV-1), and macrophages can also be infected by HIV-1 [2]. Both cell types are thought to be essential for the body’s defense against *Mtb* infection [3]. People infected with HIV-1 have a significantly lower absolute CD4^+^ T-cell count, which is strongly linked to an increased risk of TB [4]. The depletion of CD4^+^ T cells caused by HIV-1 infection has been linked to poor granuloma formation and a higher bacterial load [5]. HIV-1 infection has been reported to inhibit macrophage apoptosis in response to *Mtb* infection, potentially through HIV-1 Nef inhibition of tumor necrosis factor alpha (TNF-α) production [6, 7]. Since macrophage apoptosis is a key part of the innate immune response to inhibit intracellular *Mtb* growth, HIV-1 infection is thought to facilitate *Mtb* infection. Conversely, *Mtb* infection may result in increased HIV-1 viral loads in the blood and the site of coinfection, which can worsen HIV-1 disease progression [8–11].

Despite great achievement in study of the immunopathology of TB or HIV-1–TB coinfection, the exact changes in immune-cell subsets and the involvement of unidentified cell types in the immune response to *Mtb* or HIV-1 remain largely unknown. Single-cell RNA sequencing (scRNA-seq) is a powerful tool for studying the response of host immune cells to infection, including the characterization of novel cell subsets, cell heterogeneity, and virus–host interactions [12]. Owing to challenges in sample collection, most studies of host immunity against infection have mainly focused on systemic immune responses in peripheral blood rather than local immune responses in the lungs. Host immune response in the lungs is essential in containing the growth of *Mtb* and preventing the progression of the disease. A comprehensive analysis of the pulmonary immune system is critical for gaining a better understanding of the pathogenesis of HIV-1–TB coinfection.

By using scRNA-seq, we obtained a comprehensive view of the immunological response in bronchoalveolar lavage fluid (BALF) from people with HIV-1–TB coinfection and people with TB. Our research depicted a detailed transcriptomic profile of bronchoalveolar immune cells and highlighted the significantly different immune responses between individuals with HIV-1–TB and those with TB, which will help us to comprehend the protective and pathogenic immune responses of the diseases more effectively.

## METHODS

### Ethics Approval

All BALF samples were collected as part of routine diagnostics. This study was conducted with the approval of the Shenzhen Third People's Hospital Ethical Committee (Reference No. 2021–016), and informed consent was obtained from each participant. All the methods used in this study were in compliance with the applicable guidelines and regulations.

### Human Subjects and Sample Collection

Bronchoscopy was performed on people with suspected TB to diagnose TB. To ensure the quality of single-cell sequencing, BALF samples were immediately processed after collection (see detailed processing in the Supplementary Methods). People with active TB were diagnosed based on clinical symptoms and radiological signs as described previously [13], and had positive *Mtb* results from culture or GeneXpert. HIV-1 infection was diagnosed based on HIV-1 RNA levels. Once the diagnosis results became available, only BALF samples from patients with confirmed TB or HIV-1–TB were further subjected to construct single-cell libraries and sequencing. Therefore, it should be note that the number of collected BALF samples was much larger than the number ultimately presented in the study. Finally, a total of 10 participants with active TB alone and 9 participants with HIV-1–TB coinfection were recruited for this study. Supplementary Table 1 contains the detailed characteristics of participants. The methods for scRNA-seq, data processing, and bioinformatic analysis are detailed in the Supplementary Methods.

## Supplementary Material

jiae042_Supplementary_Data

## Figures and Tables

**Figure 1.**
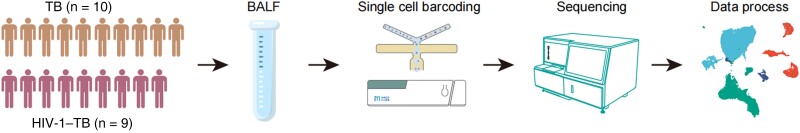
Overview of the study design and experimental workflow.

**Figure 2.**
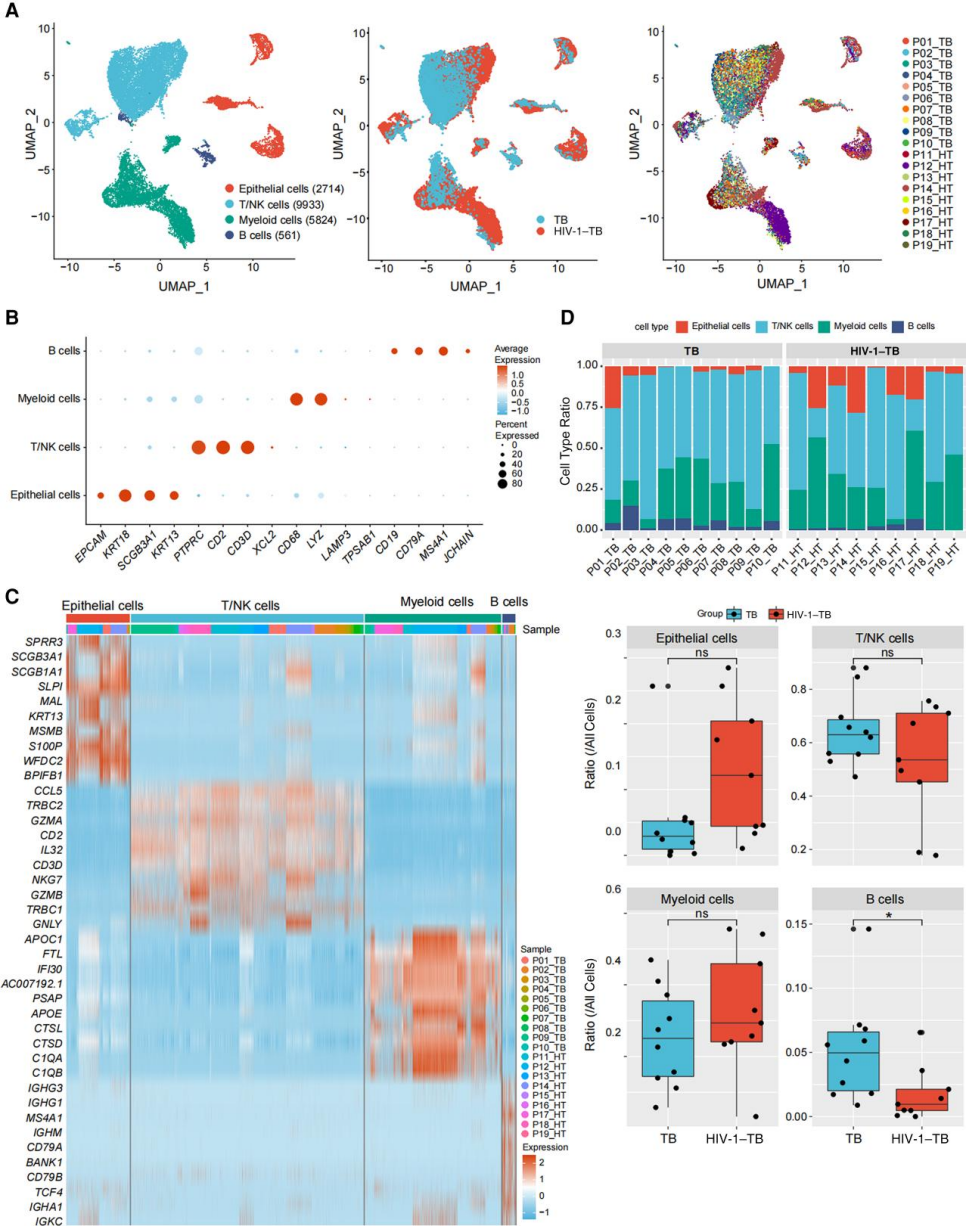
Outline of cell composition in bronchoalveolar lavage fluid (BALF) from participants with human immunodeficiency virus type 1 (HIV-1)–tuberculosis (TB) coinfection and participants with TB by single-cell RNA sequencing. *A*, Uniform manifold approximation and projection (UMAP) of 19 058 single cells from participants with HIV-1–TB coinfection (n = 9) or TB (n = 10) are color-coded by cell type, group, and sample ID, respectively. The number of cells is indicated in each cluster. *B*, Dot plots show the expression levels of marker genes in each cell type. The blue to red color indicates low to high expression levels and the size of the dots indicates the percentage of cells expressing the gene. *C*, Heatmaps show the differentially expressed genes in various cell clusters of BALF. The gene names and their corresponding cell types are listed to the left and top, respectively. *D*, Fraction of each cell type in each sample and proportions of cell types between the HIV-1–TB and TB groups. The Wilcoxon test was used to analyze the differences between 2 groups. *P < .05; ns, not significant.

**Figure 3.**
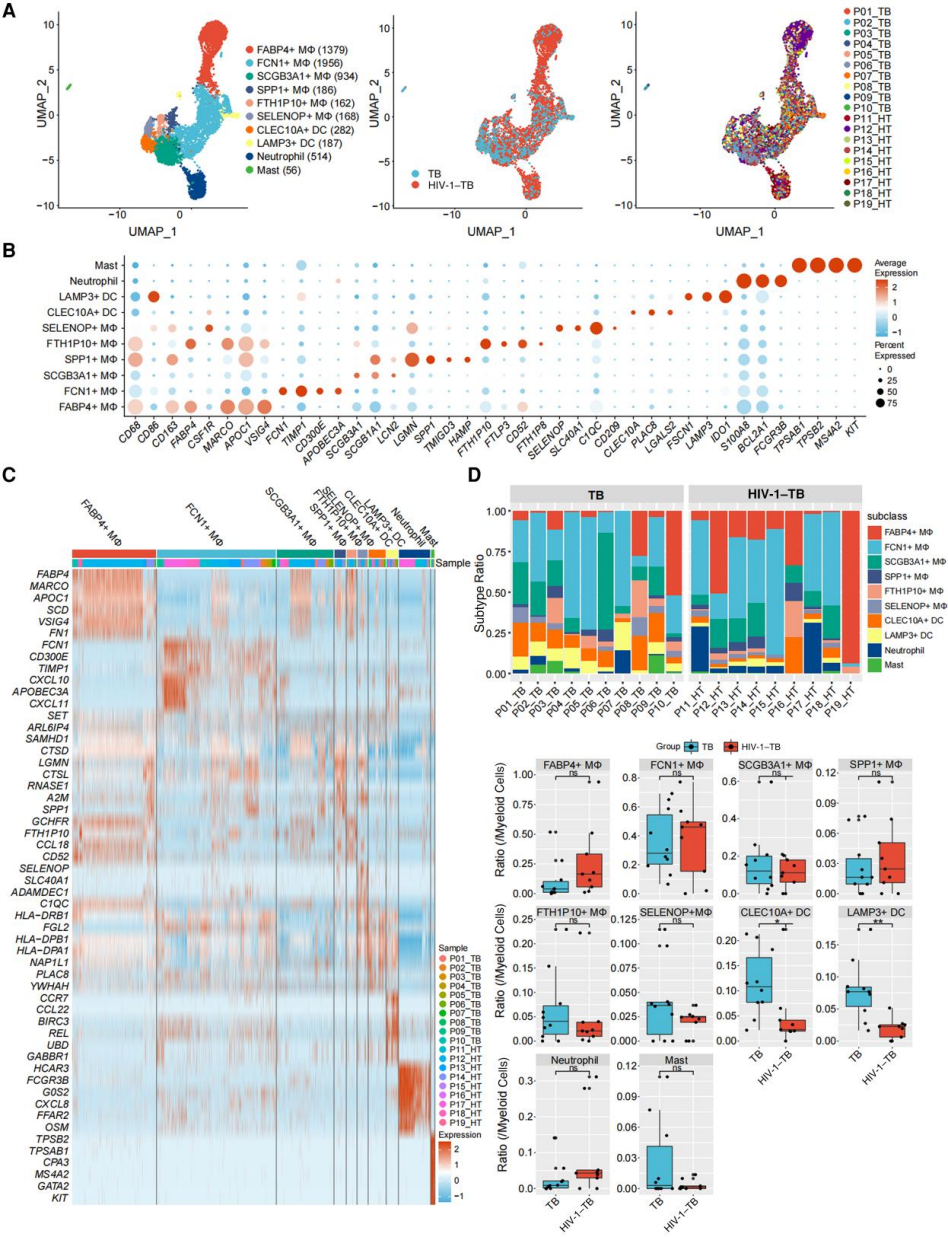
Subclustering of myeloid cells in bronchoalveolar lavage fluid (BALF) from human immunodeficiency virus type 1 (HIV-1–TB) coinfection and tuberculosis (TB) groups. *A*, Uniform manifold approximation and projection (UMAP) of myeloid cells from HIV-1–TB coinfection and TB patients are color-coded by cell clusters, cell types, groups, and sample ID, respectively. The number of cells is indicated in each cluster. *B*, Dot plots show the expression levels of marker genes in each cell subset. The blue to red indicates low to high expression levels and the size of the dots indicates the percentage of cells expressing the gene. *C*, Heatmaps show the differentially expressed genes in myeloid cell subsets of BALF. The gene names and their corresponding cell types are listed to the left and top, respectively. *D*, Fraction of each cell type in each sample and proportions of myeloid cell subsets between the 2 groups. The Wilcoxon test was used to analyze the differences between 2 groups. Significance levels are indicated as *P < .05, **P < .01,; ns, not significant.

**Figure 4.**
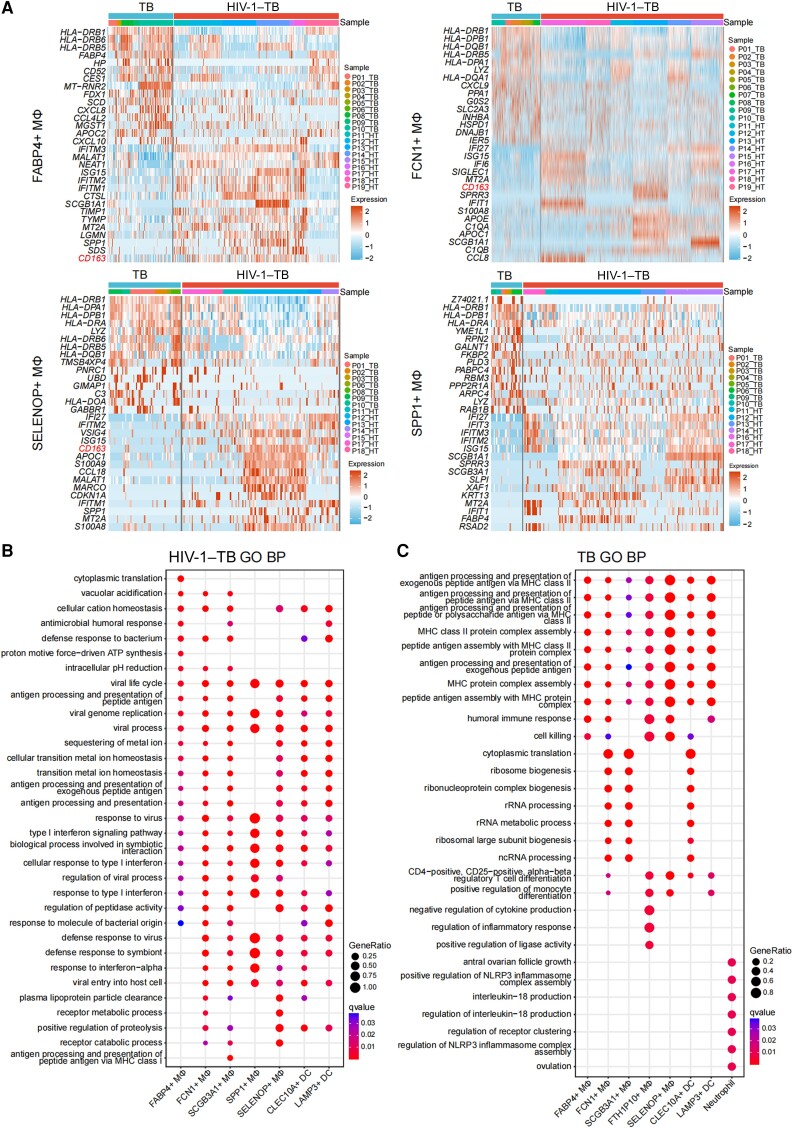
Analysis of the transcriptomic alterations of each macrophage subset between 2 groups. *A*, Heatmaps show the top 15 differentially expressed genes (DEGs) of macrophage subsets between 2 groups. The gene names and their corresponding groups are listed to the left and top, respectively. Bubble plots show the top 20 Gene Ontology biological process enrichment terms for upregulated DEGs in the human immunodeficiency virus type 1 (HIV-1)–tuberculosis (TB) group (*B*) and TB group (*C*). Abbreviations: BP, biological process; DC, dendritic cell; GO, Gene Ontology; HIV-1, human immunodeficiency virus type 1; MHC, major histocompatibility complex; TB, tuberculosis.

**Figure 5.**
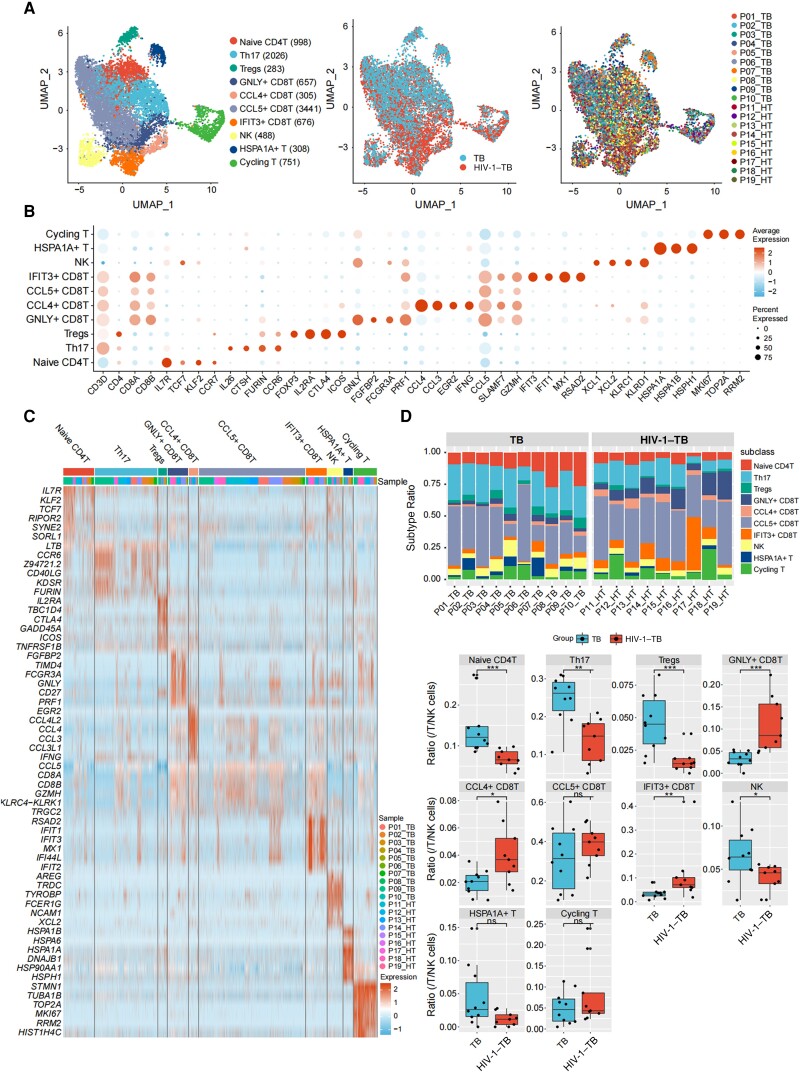
Subclustering of T/natural killer (NK) cells in bronchoalveolar lavage fluid (BALF) from participants with human immunodeficiency virus type 1 (HIV-1)–tuberculosis (TB) coinfection or TB. *A*, Uniform manifold approximation and projection (UMAP) of T/NK cells from participants with HIV-1–TB coinfection and those with TB were color-coded by cell cluster, cell type, group, and sample ID, respectively. The number of cells are indicated in each cluster. *B*, Dot plots show the expression levels of marker genes in each cell subset. Blue to red indicates low to high expression levels and the size of the dots indicates the percentage of cells expressing the gene. *C*, Heatmaps show the differentially expressed genes in various T/NK subsets of BALF. The gene names and their corresponding cell types are listed to the left and top, respectively. *D*, Fraction of T/NK subsets in each sample and proportions of T/NK subsets between the 2 groups. The Wilcoxon test was used to analyze the differences between 2 groups. Significance levels are indicated as *P < .05, **P < .01, ***P < .001; ns, not significant.

**Figure 6.**
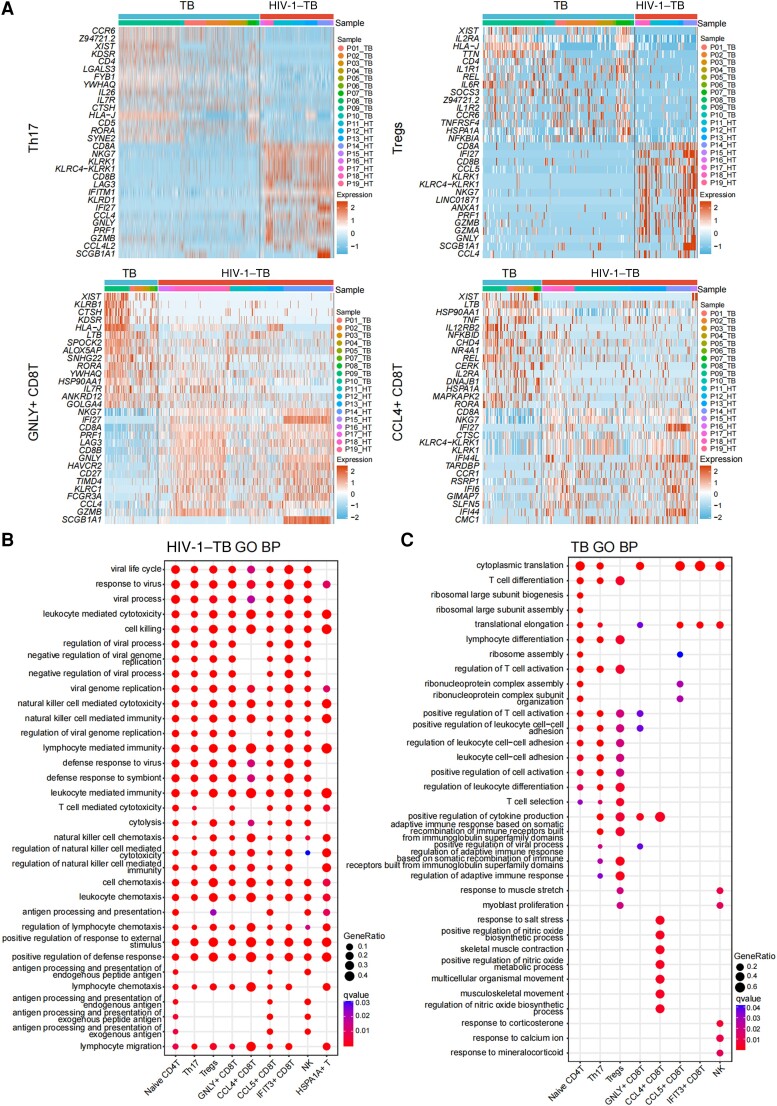
Analysis of the transcriptomic alterations of CD4 and CD8 T-cell subsets between 2 groups. *A*, Heatmaps show the top 15 differentially expressed genes (DEGs) of each T-cell subset between 2 groups. The gene names and their corresponding groups are listed to the left and top, respectively. Bubble plots show the top 20 Gene Ontology biological process enrichment terms for upregulated DEGs in the human immunodeficiency virus type 1 (HIV-1)–tuberculosis (TB) coinfection group (*B*) and TB group (*C*). Abbreviations: BP, biological process; GO, Gene Ontology; HIV-1, human immunodeficiency virus type 1; NK, natural killer; TB, tuberculosis.

## Data Availability

The data that support the findings of this study have been deposited into the CNGB Sequence Archive [14] of the China National GeneBank DataBase [15] with accession number CNP0004445.

## RESULTS

### Single-Cell Atlas of BALF in TB and HIV-1–TB Coinfection

Ten people with pulmonary TB (6 male, 4 female) and 9 with HIV-1–TB coinfection (6 male, 3 female) were recruited for scRNA-seq (Figure 1). None of the participants had received anti-TB treatment or antiretroviral treatment before sampling. The mean age of participants in the HIV-1–TB and TB groups was 44 and 43.2 years, respectively. The clinical data of all participants can be found in Supplementary Table 1. Cells were isolated from the collected BALF samples and subjected to scRNA-seq analysis. After quality control, a total of 19 058 cells were included for downstream analysis (Supplementary Table 2). Four major cell types were identified based on the expression levels of canonical marker genes without any patient bias. These cell types included T/natural killer (NK) cells (CD3D^+^CD3E^+^NKG7^+^), B cells (CD79A^+^CD79B^+^MS4A1), myeloid cells (CD68^+^LYZ^+^), and epithelial cells (SCGB1A1^+^EPCAM^+^KRT17^+^) (Figure 2A and 2B, Supplementary Figure 1*A* and 1*B*). In addition to the canonical cell-type marker genes, we also identified numerous highly expressed gene markers specific to each cell type as shown in the heatmap (Figure 2C).

After analyzing the percentage of each cell type in total cells, we found that T/NK cells had the highest average of 55%, followed by myeloid cells with an average of 29%, and B cells with an average of 3.5% (Figure 2D). Additionally, the TB and HIV-1–TB groups had similar frequencies of T/NK cells and myeloid cells (Figure 2D). However, the frequency of B cells was significantly higher in the TB group than in the HIV-1–TB group (Figure 2D). It is worth mentioning that B cells were rare in participants with HIV-1–TB coinfection, accounting for only an average of 1.7% of total cells.

### Ten Myeloid Cell Types Identified in BALF Samples Using scRNA-seq

The myeloid cells were reclassified into 10 subsets based on their distinctive gene expression profiles (Figure 3*A*). Among these clusters, 6 were identified as macrophages due to their high expression of the macrophage-associated gene CD68 (Figure 3*B* and 3*C*, Supplementary Figure 2*A* and 2*B*). These macrophages were defined as FABP4^+^ MΦ subset, FCN1^+^ MΦ subset, SCGB3A1^+^ MΦ subset, SPP1^+^ MΦ subset, FTH1P10^+^ MΦ subset, and SELENOP^+^ MΦ subset based on the high expression characteristics of gene markers (Figure 3*B*, Supplementary Figure 2*A* and 2*B*). These highly expressed gene signatures are highly correlated with macrophage function. For instance, the FABP4^+^ MΦ population expressed high levels of *FABP4* and *MARCO*, along with *APOC1* and *CD163*, indicating its resemblance to M2-like macrophages (Figure 3*B*). The FCN1^+^ MΦ subset was marked by high expression of *FCN1*, *CD300E*, *TIMP1*, and *APOBEC3A* (Figure 3*B*) and also expressed high levels of chemokines such as *CXCL10* and *CXCL11* (Figure 3*C*). The SCGB3A1^+^ MΦ population expressed high levels of secretoglobin *SCGB3A1*, *SCGB1A1*, and *LCN2* (Figure 3*B*). The SPP1^+^ MΦ population had a high expression of *SPP1*, along with high expression of genes encoding proteases including *LGMN*, *TMIGD3*, *and HAMP* (Figure 3*B*). Notably, the SPP1^+^ MΦ subset also showed high expression of *CD163*, indicating an anti-inflammatory phenotype (Figure 3*B*). The FTH1P10^+^ MΦ population showed similarities to the FABP4^+^ MΦ subset, as they both exhibited high expression of *FABP4* and *MARCO* (Figure 3*B* and 3*C*). Additionally, the FTH1P10^+^ MΦ population showed high expression of *FTLP3*, *CD52*, and *FTH1P8* (Figure 3*B*). The SELENOP^+^ MΦ population was specifically enriched for high expression of *SELENOP*, *SLC40A1*, and *C1QC* (Figure 3*B*).

Tissue-resident alveolar macrophages and monocyte-derived interstitial macrophages play distinct roles in relation to *Mtb* infection. Alveolar macrophages tend to support *Mtb* growth, while interstitial macrophages tend to restrict *Mtb* growth [16]. To determine the preferences of macrophage subsets for infiltration or tissue residence, we utilized the AddModuleScore function in Seurat with reported gene sets to assess the origin of different macrophage subsets [17]. According to the score, the FABP4^+^ MΦ subset, SCGB3A1^+^ MΦ subset, SPP1^+^ MΦ subset, and FTH1P10^+^ MΦ subset may originate from alveolar macrophages, while the FCN1^+^ MΦ subset and FTH1P10^+^ MΦ subset may originate from the periphery (Supplementary Figure 2*C*).

Two subsets of dendritic cells (DCs), CLEC10A*^+^* DCs and LAMP3^+^ DCs, were identified based on canonical marker genes. The CLEC10A^+^ DC population was characterized by elevated expression of *CLEC10A*, *PLAC8*, and *LGAL32* (Figure 3*B*). The LAMP3^+^ DC was characterized by high expression of *CD86*, *LAMP3*, *FSCN1*, and *IDO1* (Figure 3*B*). Furthermore, we identified a neutrophil cell population with high expression of *S100A8*, *BCL2A1*, and *FCGR3B* and a mast cell subset with high levels of *TPSAB1*, *TPSB2*, *MS4A2*, and *KIT* (Figure 3*B*). Moreover, we present a heatmap that shows many other gene markers specific to each myeloid cell subpopulation that may be involved in the functional diversification of myeloid cell subpopulations (Figure 3*C*).

### Depletion of DCs in the HIV-1–TB Coinfection Group Versus the TB Group

Among the myeloid cells, macrophages comprised the majority (80%), followed by DCs (13%), neutrophil cells (5.6%), and mast cells (1.4%) (Figure 3*D*). Of the 10 subsets, the FCN1^+^ MΦ subset had the highest average proportion of 36%, followed by FABP4^+^ MΦ with an average of 18% and SCGB3A1^+^ MΦ with an average of 14% (Figure 3*D*). However, there were no significant differences in the frequencies of any MΦ subsets between the 2 groups, both in total myeloid cells and in total immune cells (Figure 3*D*, Supplementary Figure 3*A*). Similarly, neutrophils and mast cells also showed similar frequencies between the 2 groups, both in total myeloid cells and in total immune cells (Figure 3*D*, Supplementary Figure 3*A*). However, the HIV-1–TB group had lower frequencies of CLEC10A^+^ DC and LAMP3^+^ DC subsets in total myeloid cells than the TB group (Figure 3*D*). Furthermore, the HIV-1–TB group also had a significantly lower frequency of CLEC10A^+^ DCs in total immune cells (Supplementary Figure 3*A*). These results suggest that HIV-1 infection may have depleted DCs in patients with TB.

### Analysis of the Transcriptomic Alterations of Myeloid Cells Between 2 Groups

We identified hundreds of genes that were significantly expressed in each macrophage subset between the 2 groups (Supplementary Tables 3–14), indicating that HIV-1 infection has a significant effect on macrophage functions. The heatmap displays the top 15 differentially expressed genes (DEGs) in each macrophage subset between the 2 groups (Figure 4*A* and Supplementary Figure 3*B*). Among them, CD163 was significantly upregulated in FABP4^+^ MΦ, FCN1^+^ MΦ, and SELENOP^+^ MΦ subsets in the HIV-1–TB group, suggesting that these macrophage subsets tend to exhibit M2-like anti-inflammatory phenotype in people with HIV-1–TB coinfection (Figure 4*A*). Gene Ontology (GO) term enrichment analysis revealed that the upregulated DEGs in each macrophage subset in the HIV-1–TB group were mainly associated with cellular cation homeostasis, virus replication, host defense to virus, and type I interferon responses (Figure 4*B*). On the other hand, the upregulated DEGs in each macrophage subset in the TB group were mainly related to major histocompatibility complex (MHC) protein complex assembly, peptide antigen assembly with MHC protein complex, and humoral immune response (Figure 4*C*).

### Eleven T/NK Cell Subsets Identified in BALF Samples Using scRNA-seq

The total T/NK cells were reclustered into 11 subsets (Figure 5*A*). Among these subsets, 10 expressed high levels of *CD3D*, a canonical T-cell gene marker (Figure 5*B* and 5*C*, Supplementary Figure 4*A* and 4*B*). The remaining cluster was identified as NK cells based on its high expression of typical NK cell genes such as *KLRC1* and *KLRD1* (Figure 5*B* and 5*C*). These NK cells were also characterized with high levels of *GNLY*, *XCL1*, and *XCL2* (Figure 5*B* and 5*C*). Three CD4 T-cell subsets were defined as naive CD4 T cells, Th17 cells, and Treg cells based on canonical cell gene markers (Figure 5*B* and 5*C*, Supplementary Figure 4*A* and 4*B*). Naive CD4 T cells were characterized by high expression of *IL7R*, *TCF7*, *CCR7*, and *KLF2*; Th17 cells by high expression of *IL26*, *CTSH*, and *CCR6*; and Treg cells by high expression of *FOXP3*, *IL2RA*, and *CTLA4* (Figure 5*B* and 5*C*). Four CD8 T-cell subsets were identified based on the high expression of *CD8A* and *CD8B*, which were all effector cells due to their *NKG7*, *GZMB*, and *GZMH* expression (Figure 5*B* and 5*C*). The 4 CD8 effector T subsets were defined as GNLY^+^ CD8 T subset, CCL4^+^ CD8 T subset, CCL5^+^ CD8 T subset, and IFIT3^+^ CD8 T subset. The GNLY^+^ CD8 T subset highly expressed cytotoxic genes including *GNLY*, *FCGR3A*, and *PRF1*, indicating its cytotoxic state (Figure 5*B* and 5*C*). The CCL4^+^ CD8 T subset was characterized by high levels of chemokines including *CCL4*, *CCL3*, *EGR2*, and *IFNG*, suggesting it played a proinflammatory role (Figure 5*B* and 5*C*). The CCL5^+^ subset was marked by high expression levels of cytotoxic signals, including *CCL5* and *GZMH* (Figure 5*B* and 5*C*). The IFIT3^+^ subset was characterized by high levels of interferon inducible genes such as *IFIT3*, *IFIT1*, and *RSAD2*, suggesting a robust type I interferon response (Figure 5*B* and 5*C*). A subpopulation characterized by the high expression of genes encoding heat shock proteins including *HSPA1A*, *HSPA1B*, and *HSPH1*, but not *CD4* and *CD8*, was classified as the HSPA1A^+^ T-cell subset (Figure 5*B* and 5*C*). Additionally, a subset with low expression of *CD4* and *CD8*, but highly expressed *MKI67*, *TOP2A*, and *RRM2*, was identified as the cycling T subset (Figure 5*B* and 5*C*).

### CD4^+^ T Cells Depleted and CD8^+^ T Cells Expanded in Patients With HIV-1–TB Coinfection Compared to Those With TB

The frequency of CD4 T cells was much lower than that of CD8 T cells in the HIV-1–TB group, which is consistent with the clinical test results showing a lower frequency of CD4 T cells compared to CD8 T cells in the participants’ blood (Figure 5*D*, Supplementary Table 1). In both total T/NK cells and total immune cells, the frequencies of all 3 CD4 T-cell subsets (naive CD4 T cells, Th17 cells, and Treg cells) were significantly lower in the HIV-1–TB group compared to the TB group (Figure 5*D*, Supplementary Figure 5*A*). In contrast, the proportions of all 4 CD8 T-cell subsets (GNLY^+^ CD8 T cells, CCL4^+^ CD8 T cells, CCL5^+^ CD8 T cells, and IFIT3^+^ CD8 T cells) were significantly higher in the HIV-1–TB group compared to the TB group (Figure 5*D*). Additionally, the proportions of the IFIT3^+^ CD8 T-cell and GNLY^+^ CD8 T-cell subsets in immune cells were also significantly higher in the HIV-1–TB group than in the TB group (Supplementary Figure 5*B*). However, the frequency of CCL4^+^ CD8 T-cell subsets in total immune cells was not significantly different between the 2 groups (Supplementary Figure 5*A*). Notably, the frequency of NK cells in both total T/NK cells and total immune cells was significantly lower in the HIV-1–TB group compared to the TB group (Figure 5*D*, Supplementary Figure 5*B*). The remaining T-cell subsets (cycling T, unknown T, and HSPA1A^+^ T subsets) had similar frequencies in both total T/NK cells and immune cells in the 2 groups (Figure 5*D*, Supplementary Figure 5*A*). These findings suggest that HIV-1 infection depletes CD4 T and NK cells while expanding CD8 T cells in the lungs of TB patients.

### Analysis of the Transcriptomic Differences in NK, CD4, and CD8 T-Cell Subsets Between the HIV-1–TB and TB Groups

Within each analyzed T-cell subset, hundreds of genes were found to be significantly expressed between the 2 groups (Supplementary Tables 15–30). It is noteworthy that the number of upregulated DEGs in participants with HIV-1–TB coinfection patients was much greater than that in TB patients for each analyzed subset (Supplementary Tables 15–30). The heatmap displayed the top 15 upregulated or downregulated DEGs in each analyzed T-cell subset between the 2 groups (Figure 6*A*, Supplementary Figure 5*B*). All analyzed subsets showed an elevated expression of cytotoxic genes, such as *GNLY*, *GZMA/B/H/K*, and *NKG7*, indicating a higher level of cytotoxicity in the HIV-1–TB group compared to the TB group (Figure 6*A*, Supplementary Figure 5*B*). In individuals with HIV-1–TB coinfection, both CD4 and CD8 T cells showed higher expression of IFN-inducible genes such as *IFI27*, *IFI44*, *IFI44L*, and *ISG15* (Figure 6*A*, Supplementary Figure 5*B*), suggesting greater antiviral activity in these cells compared to those in TB patients. GO term enrichment analysis revealed that the upregulated DEGs in each CD4 and CD8 T-cell subsets in the HIV-1–TB group were mainly associated with virus replication, host responses to virus, leukocyte-mediated cytotoxicity, and cell killing (Figure 5*B*). In contrast, the upregulated DEGs in CD4 T-cell subsets were mainly enriched in cytoplasmic translation, T-cell differentiation, T-cell activation, leukocyte cell–cell adhesion, and leukocyte differentiation, while the DEGs in each CD8 T-cell and NK cell subset had distinct enrichment of GO terms (Figure 6*C*). These findings suggest that HIV-1 infection not only affects the frequency of CD4 and CD8 T cells, but also influences their functions.

## DISCUSSION

There are limited information on the local cellular immune responses in the lungs of individuals coinfected with HIV-1–TB, due to the challenges in obtaining lung samples. To gain insight into the immune responses in lungs of individuals with HIV-1–TB coinfection, as well as those with TB only, we collected BALF samples from 10 patients with active TB and 9 with HIV-1–TB coinfection (Figure 1). ScRNA-seq was conducted on BALF samples to explore the transcriptomic landscape of immune cells.

We identified 6 distinct macrophage subsets in BALF samples, each with its own transcriptomic profile, indicating that these subsets may serve different purposes in response to microbial infections in lungs (Figure 3*A*–*C*). The majority of myeloid cell subsets displayed no significant difference in frequency between the HIV-1–TB coinfection group and the TB group (Figure 3*D*). However, we did find a number of DEGs in macrophage subsets between the 2 groups (Figure 4*A* and Supplementary Figure 3*B*). Macrophages in HIV-1–TB patients exhibited a stronger antiviral activity, while macrophages in TB patients demonstrated a more prominent antimicrobial humoral response. DCs were the only myeloid cell subset to display different frequencies between people with HIV-1–TB coinfection and those with TB (Figure 3*D*). This decrease in DCs in people with HIV-1 may increase their susceptibility to TB.

A drastic decrease in CD4^+^ T cells is a characteristic of progressive HIV-1 infection [18]. It has been suggested that Th17 cells are essential for the immune system to protect against tuberculosis, as they are involved in the induction of Th1 cells to activate macrophages, the recruitment of these cells to the sites of infection, and the formation of granuloma [19–21]. A decrease in the frequency of BALF Th17 cells in HIV-1–TB–coinfected patients has been observed, which is consistent with other studies that have demonstrated a depletion of Th17 cells in the gut of HIV-positive individuals [22]. In people with HIV-1–TB coinfection, the decrease in Th17 cell subcluster frequency can be damaging to *Mtb* infection but may help to reduce inflammation related to lung injury. During the initial stages of tuberculosis, *Mtb* causes an increase in Treg cells in the lungs, which delays the start of adaptive immunity [23, 24]. During chronic infection, Treg cells can be beneficial to the lungs by moderating inflammation [25]. We determined that the frequencies of Tregs in the lungs of participants with HIV-1–TB coinfection were dramatically lower in comparison to those with TB, which is contrary to other studies, which highlighted that HIV infection leads to an enlargement of Treg frequency in total T cells in peripheral blood in both acute and chronic phases of the infection [26–29]. The remarkable depletion of Tregs may indicate a severe imbalance of anti-inflammatory and proinflammatory response in people with HIV-1–TB coinfection. Expansion of CD8 T cells can be observed in both peripheral blood and BALF in people with HIV-1 [30–33]. Animal models have demonstrated that CD8 T cells are capable of providing host protection against *Mtb* [34]. We discovered 4 CD8 effector T-cell subsets with high expression of cytotoxic genes in BALF samples, and these populations were significantly more frequent in the HIV-1–TB group compared to the TB group (Figure 5*D*, Figure 6*A*, Supplementary Figure 5*B*). The expansion of CD8 T cells in lungs may contribute to contain *Mtb* in people with HIV-1–TB coinfection.

Numerous studies have provided evidence that HIV-1 not only depletes the number of CD4^+^ T cells, but also weakens the T cells’ capacity to fight *Mtb* [35–38]. In this study, we observed numerous DEGs in both CD4 and CD8 T-cell subsets between the HIV-1–TB and TB groups (Figure 6*A*, Supplementary Figure 5*B*). In individuals with HIV-1–TB coinfection, CD4 and CD8 T cells exhibited enhanced antiviral activity, as evidenced by their higher expression of interferon-inducible genes like *IFI27*, *IFI44*, *IFI44L*, and *ISG15*, compared to TB patients (Figure 6*A*, Supplementary Figure 5*B*). These findings highlight that HIV-1 infection not only impacts the number of T cells in the lungs of people with TB, but also their functionality.

## Supplementary Data

Supplementary materials are available at *The Journal of Infectious Diseases* online (http://jid.oxfordjournals.org/). Supplementary materials consist of data provided by the author that are published to benefit the reader. The posted materials are not copyedited. The contents of all supplementary data are the sole responsibility of the authors. Questions or messages regarding errors should be addressed to the author.

## Notes

***Author contributions.*** All authors were involved in the design, implementation, and composition of the manuscript. All authors have read and agreed to the final version of the article.

***Acknowledgments.*** We are profoundly grateful for the nurses and doctors who aided in sample collection.

***Financial support*.** This work was supported by the National Key Research and Development Plan (No. 2021YFA1300902); the National Natural Science Foundation of China (No. 82170009); the Guangdong Science Fund for Distinguished Young Scholars (No. 0620220214); the State Key Laboratory of Respiratory Diseases Open Project (No. SKLRD-OP-202324); the Shenzhen Scientific and Technological Foundation (No. KCXFZ20211020163545004, RCJC20221008092726022, LCYX20220620105200001); the Guangxi Scientific and Technological Foundation (No. AA22096027); and China National GeneBank and the Shenzhen Key Laboratory of Single-Cell Omics (ZDSYS20190902093613831). Funding to pay the Open Access publication charges for this article was provided by the National Natural Science Foundation of China (No. 82170009).
