# Supplementary material for: Uncovering the Bronchoalveolar Single-Cell Landscape of Patients With Pulmonary Tuberculosis With Human Immunodeficiency Virus Type 1 Coinfection
Source: J Infect Dis. 2024 Feb 27;230(3):e524–35. doi: 10.1093/infdis/jiae042 (PMC11420811; doi:10.1093/infdis/jiae042)
Supplement: jiae042_Supplementary_Data [file jiae042_supplementary_data.zip › supplementary methods(clean).docx]

**Uncovering the bronchoalveolar single-cell landscape of pulmonary tuberculosis patients with HIV-1 co-infection**

Guohui Xiao^1, #^, Waidong Huang^2, 3, #^, Yu Zhong^2, #^, Min Ou^1,#^, Taosheng Ye^1^, Zhifeng Wang^2^, Xuanxuan Zou^2, 3^, Feng Ding^1^, Yuan Yang^2^, Zhe Zhang^4^, Chuanyu Liu^2, 6^, Aimei Liu^5^, Longqi Liu^2, 6^, Shuihua Lu^1,^ *, Guoliang Zhang^1,^*, Liang Wu^2, 7^ *

1. National Clinical Research Center for Infectious Diseases, Shenzhen Third People's Hospital, Southern University of Science and Technology, Shenzhen, 518112, China

2. BGI Research, Shenzhen, 518083, China

3. College of Life Sciences, University of Chinese Academy of Sciences, Beijing 100049, China

4. MGI Tech, Shenzhen, 518083, China

5. Department of Tuberculosis, Guangxi Chest Hospital, Liuzhou, 545005, China

6. BGI Research, Hangzhou, 310030, China

7. BGI Research, Chongqing, 401329, China

# These authors contributed equally to this work.

*Correspondence author

Dr. Liang Wu, wuliang@genomics.cn

Dr. Guoliang Zhang, szdsyy@aliyun.com

Dr. Shuihua Lu, lushuihua66@126.com

**Methods and materials**

**Sample processing**

BALF were obtained from participants and filtered through a 40-µm nylon cell strainer to remove clumps and debris. The red blood cells were lysed and washed with ice-cold PBS. Dead cells were removed from the suspension using the Dead Cell Removal Kit (Miltenyi Biotec) according to the manufacturer’s instructions. The entire process was completed within 30 min. And the remaining live cells were used for [single-cell isolation](about:blank).

**Single‑cell RNA library construction and sequencing**

For scRNA-seq library preparation, the DNBelab C4 system was used as previously described by Liu et al. [[1](#_ENREF_1)]. Briefly, single-cell suspensions were subjected to a series of steps, including droplet generation, emulsion breakage, mRNA capture, reverse transcription, cDNA amplification and purification, resulting in barcoded scRNA-seq libraries. Short cDNA fragments of 250-400 bp were produced through shearing and indexed sequencing libraries were made according to manufacturer instructions. The libraries were assessed with the Qubit ssDNA Assay Kit (Thermo Fisher Scientific) and Agilent 2100 Bioanalyzer. All libraries were sequenced using pair-end sequencing on the DIPSEQ T1 sequencing platform at the China National GeneBank.

**scRNA-seq data processing and annotation of cell types**

The sequencing data were processed using an open-source pipeline available at network (https://github.com/MGI-tech-bioinformatics/DNBelab_C_Series_HT_

scRNA-analysis-software). In brief, sample de-multiplexing, barcode processing, and single-cell 30 unique molecular identifier (UMI) counting were performed on all samples, using default parameters. The resulting reads were then aligned to the GRCh38 genome reference using STAR (version 2.5.3). The available cells were automatically acquired based on the UMI number distribution of each cell using the "barcodeRanks()" function of the DropletUtils tool. Subsequently, PISA was utilized to calculate the gene UMI count of cells and generate a gene x cell UMI count matrix. The resulting gene expression matrix was then used for cell clustering analysis with Seurat (version 4.1.0). Cells with less than 200 genes (UMI >0) or over 10% UMI originating from the mitochondrial genome were identified as low-quality cells. After removing low-quality cells, the gene count matrix was normalized using log1p normalization. Next, the top 3000 highly variable genes were selected to perform principal component analysis. The Harmony algorithm [[2](#_ENREF_2)] was utilized to integrate the healthy control data with our data by employing 15 principal components. Subsequently, 15 dimensions of harmony reduction were used to perform Louvain clustering and Uniform Manifold Approximation and Projection (UMAP)-based visualization using UMAP. Clusters were identified using Seurat FindClusters function, optimizing the modularity with the Louvain algorithm. The resolution parameter to control cluster granularity was set at 0.6. Differentially expressed genes (DEGs) were determined on the normalized matrix using FindAllMarkers (Wilcox test with Bonferroni correction for multiple testing; adjusted P < 0.05). Only genes detected in at least 25% of the cells within the cluster and showing a minimum 0.2-fold difference (log-scale) between the cells in the cluster and all remaining cells were tested. We further identified subpopulations and their differential genes within each major cell cluster using the same procedure mentioned previously, all starting from the UMI matrix. The cell subpopulation identities were determined based on top-ranked DEGs and previously reported related to biology. To identify cell types, known markers highly expressed in specific subsets were used. For sub-clustering of T/NK cell and myeloid cell subtypes, 10 principal components (PCs) for harmony reduction were used. The top 10 dimensions of harmony reduction were then used for clustering and UMAP visualization.

**Differential Gene Expression and Gene Ontology Analysis**

To identify differentially expressed genes (DEGs) in each subset or group, the FindAllMarkers function of the Seurat package (version 4.1.0) [[3](#_ENREF_3)] was utilized with default parameter settings (logfc.threshold = 0.25 and min.pct = 0.25). DEGs were determined using a Bonferroni-adjusted *p*-value threshold of < 0.05 and log2(fold change) ≥ 0.25. The ClusterProfiler (version 4.2.0) [[4](#_ENREF_4)] tool was then employed to conduct GO enrichment analysis on the top 200 DEGs of a specific cell type across various groups. Significance was determined using a *p*-value threshold of < 0.05 and log_2_(fold change) ≥ 0.25.

**Statistical Analysis**

The differences in cell ratio between two groups were assessed using the Wilcoxon test, with statistical significance set at a *p*-value threshold of <0.05. Significance levels were indicated as **p* < 0.05, ***p* < 0.01, ****p*< 0.001 and *****p* < 0.00001. All data analysis and presentation was done using R (version 4.1.2).

**References**

1. Liu C, Wu T, Fan F, Liu Y, Liu L: **A portable and cost-effective microfluidic system for massively parallel single-cell transcriptome profiling**. *Cold Spring Harbor Laboratory* 2019.

2. Korsunsky I, Millard N, Fan J, Slowikowski K, Raychaudhuri S: **Fast, sensitive and accurate integration of single-cell data with Harmony**. *Nature Methods* 2019, **16**(4):1-8.

3. Hao Y, Hao S, Andersen-Nissen E, Mauck WM, Satija R: **Integrated analysis of multimodal single-cell data**. *Cold Spring Harbor Laboratory* 2020.

4. T W, E H, S X, M C, P G, Z D, T F, L Z, W T, L Z *et al*: **clusterProfiler 4.0: A universal enrichment tool for interpreting omics data**. *Innovation (New York, NY)* 2021, **2**(3):100141.

**Supplementary figure legends**

**Figure S1 Displaying the marker genes for each cell type.**

1. UMAP plots show the expression of canonical cell markers in each cell type. (B) Violin plots show the scaled expression levels of canonically cell marker genes in each cell type.

**Figure S2 Displaying the marker genes for each myeloid cell subset.**

1. UMAP plots showed the expression of canonical cell markers in each myeloid cell subsets. (B) Violin plots showed the scaled expression levels of canonically cell marker genes in each myeloid cell subsets. (C) Violin plots showed the scores for determination of the preferences of macrophage subsets for infiltration or tissue residence. Significance levels were indicated as **p* < 0.05, ***p* < 0.01, ****p* < 0.001 and *****p* < 0.0001.

**Figure S3 Frequencies of each myeloid subsets in total immune cells and top 15 DEGs of myeloid subsets between two groups.**

1. The proportions of each myeloid cell subsets in total immune cells between the two groups. (B) Heatmaps show the top 15 DEGs of myeloid subsets between two groups. The Wilcoxon test was used to analyze the differences between two groups. Significance levels were indicated as **p* < 0.05, ***p* < 0.01, ****p* < 0.001.

**Figure S4 Displaying the marker genes for each T cell subset.**

1. UMAP plots show the expression of canonical cell markers in each T/NK cell subsets. (B) Violin plots show the scaled expression levels of canonically cell marker genes in each T/NK cell subsets.

**Figure S5 Frequencies of each T cell subset in total immune cells and top 15 DEGs of T cell subsets between two groups.**

1. The proportions of each T/NK subsets in total immune cells between the two groups. (B) Heatmaps show the top 15 DEGs of myeloid subsets between two groups. The Wilcoxon test was used to analyze the differences between two groups. Significance levels were indicated as **p* < 0.05, ***p* < 0.01, ****p* < 0.001.
