# Supplementary figures and images for: Uncovering the Bronchoalveolar Single-Cell Landscape of Patients With Pulmonary Tuberculosis With Human Immunodeficiency Virus Type 1 Coinfection
Source: J Infect Dis. 2024 Feb 27;230(3):e524–35. doi: 10.1093/infdis/jiae042 (PMC11420811; doi:10.1093/infdis/jiae042)

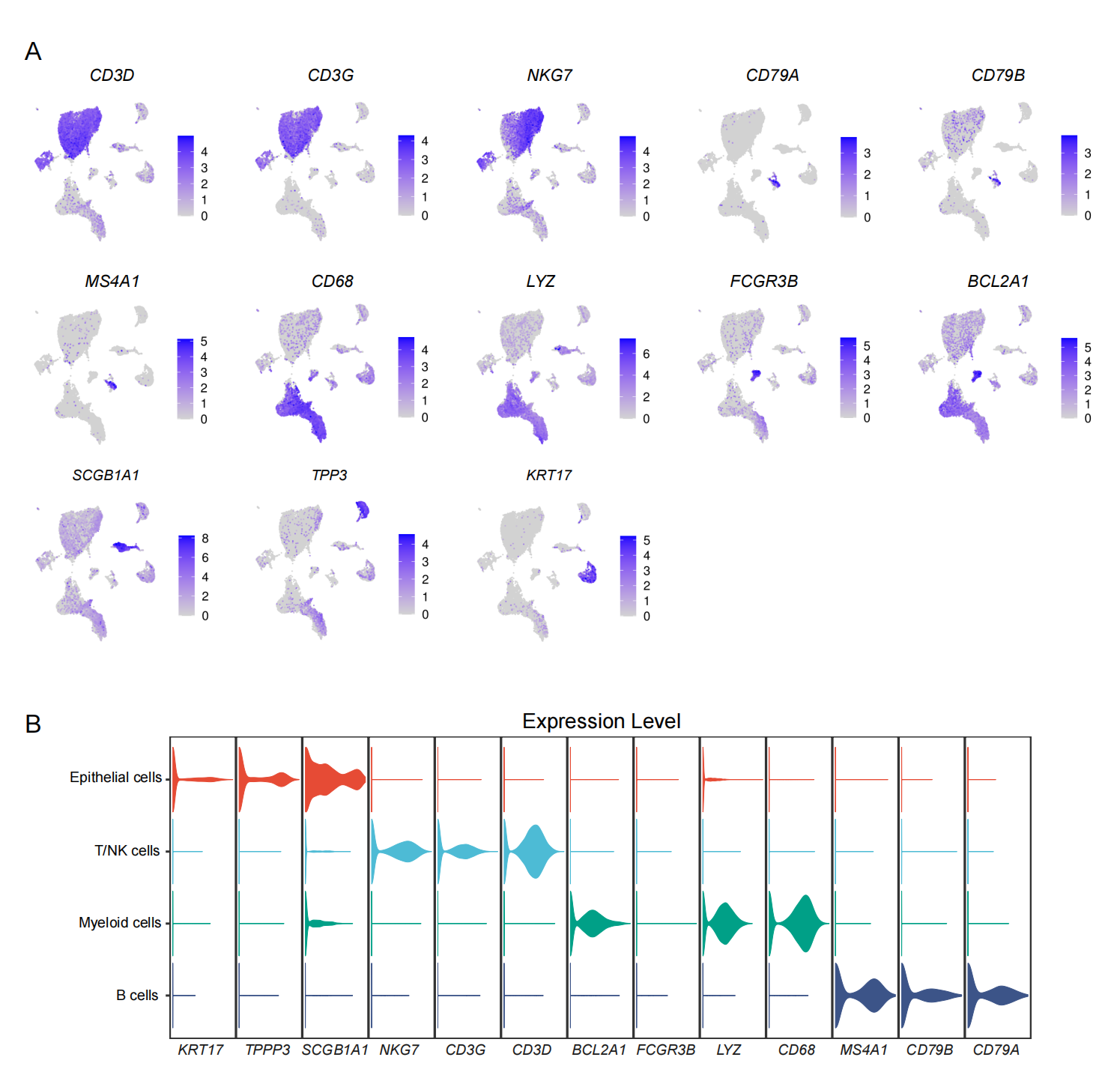

Supplement: jiae042_Supplementary_Data [file jiae042_supplementary_data.zip › FigureS1.tif]

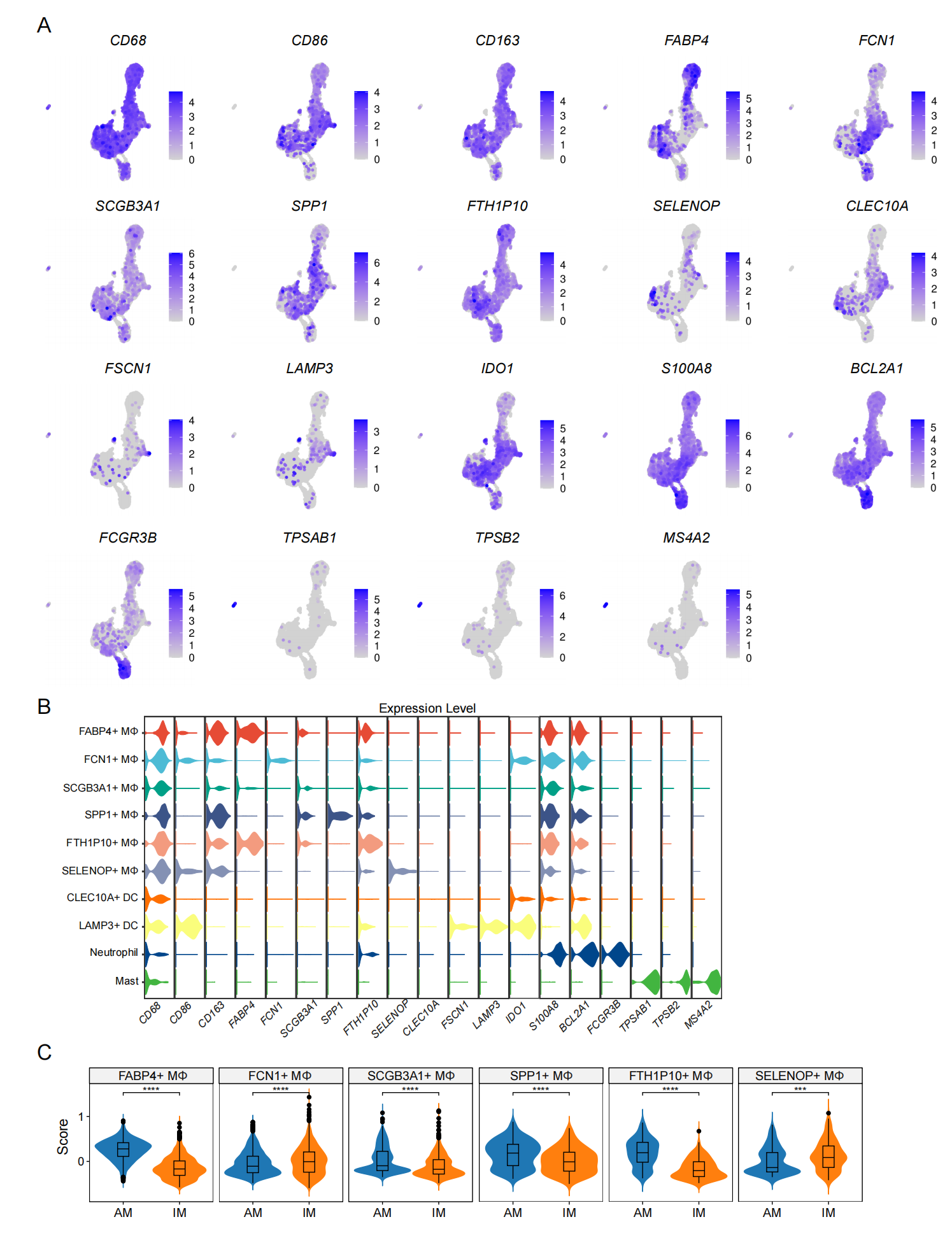

Supplement: jiae042_Supplementary_Data [file jiae042_supplementary_data.zip › FigureS2.tif]

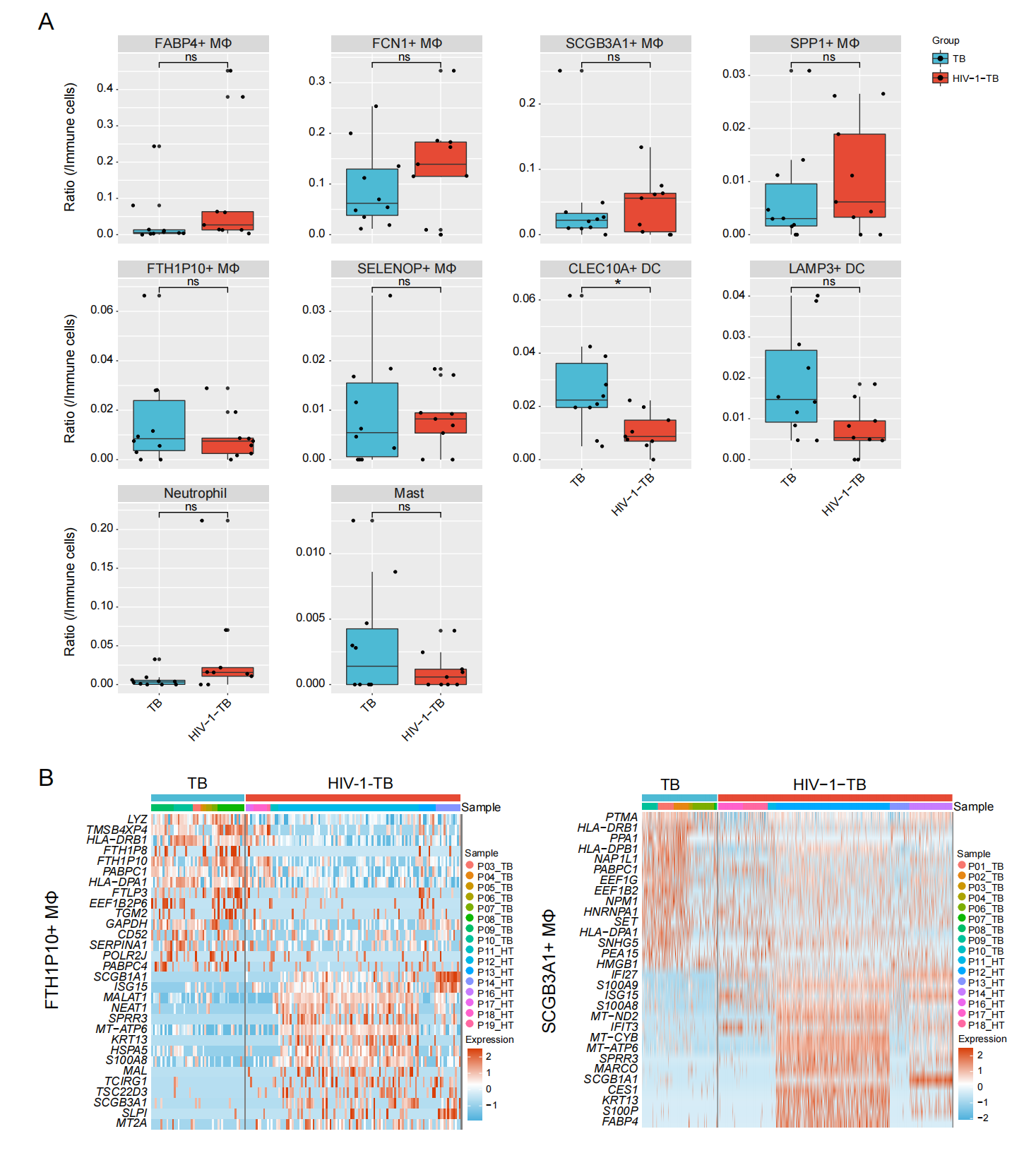

Supplement: jiae042_Supplementary_Data [file jiae042_supplementary_data.zip › FigureS3.tif]

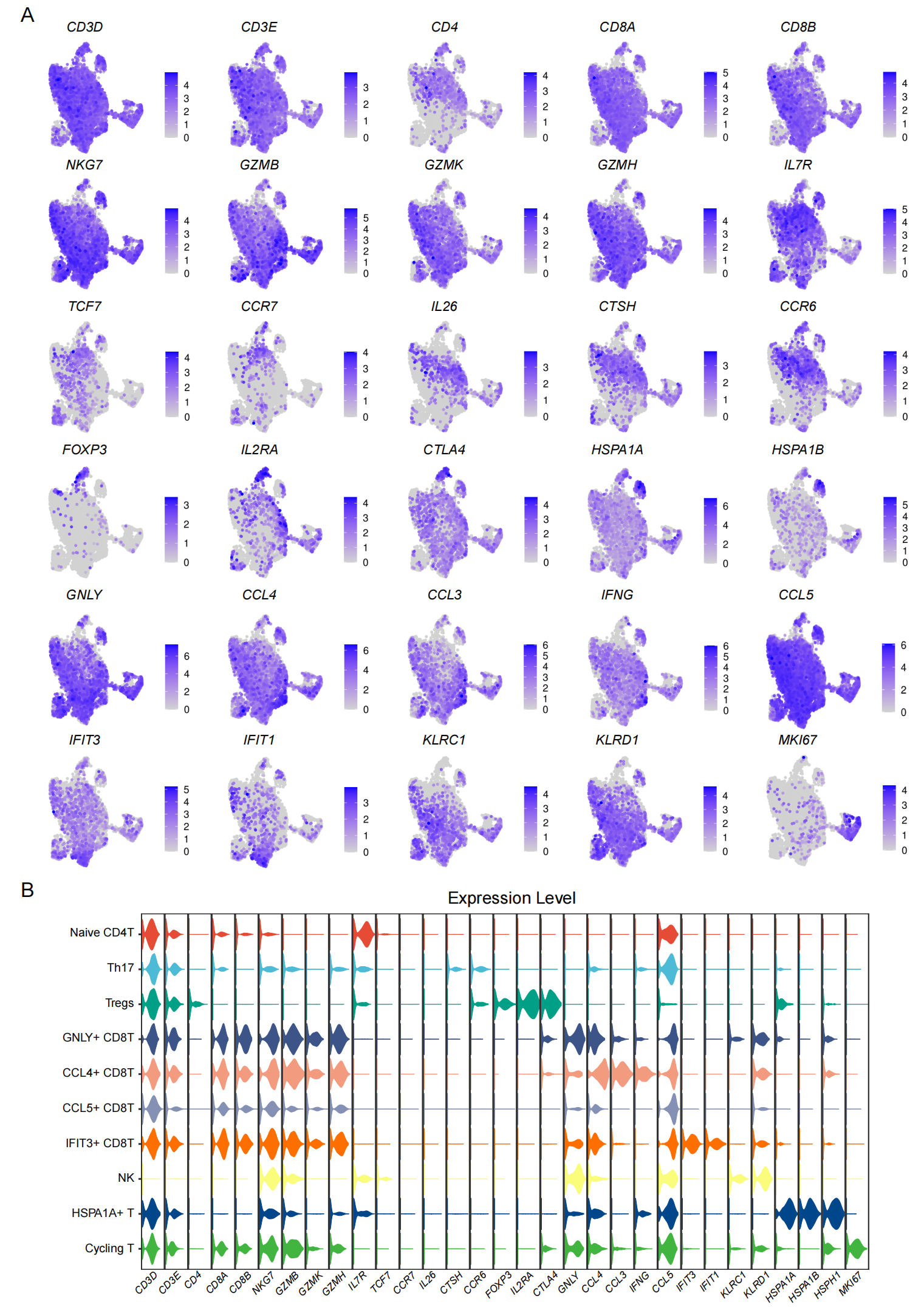

Supplement: jiae042_Supplementary_Data [file jiae042_supplementary_data.zip › FigureS4.tif]

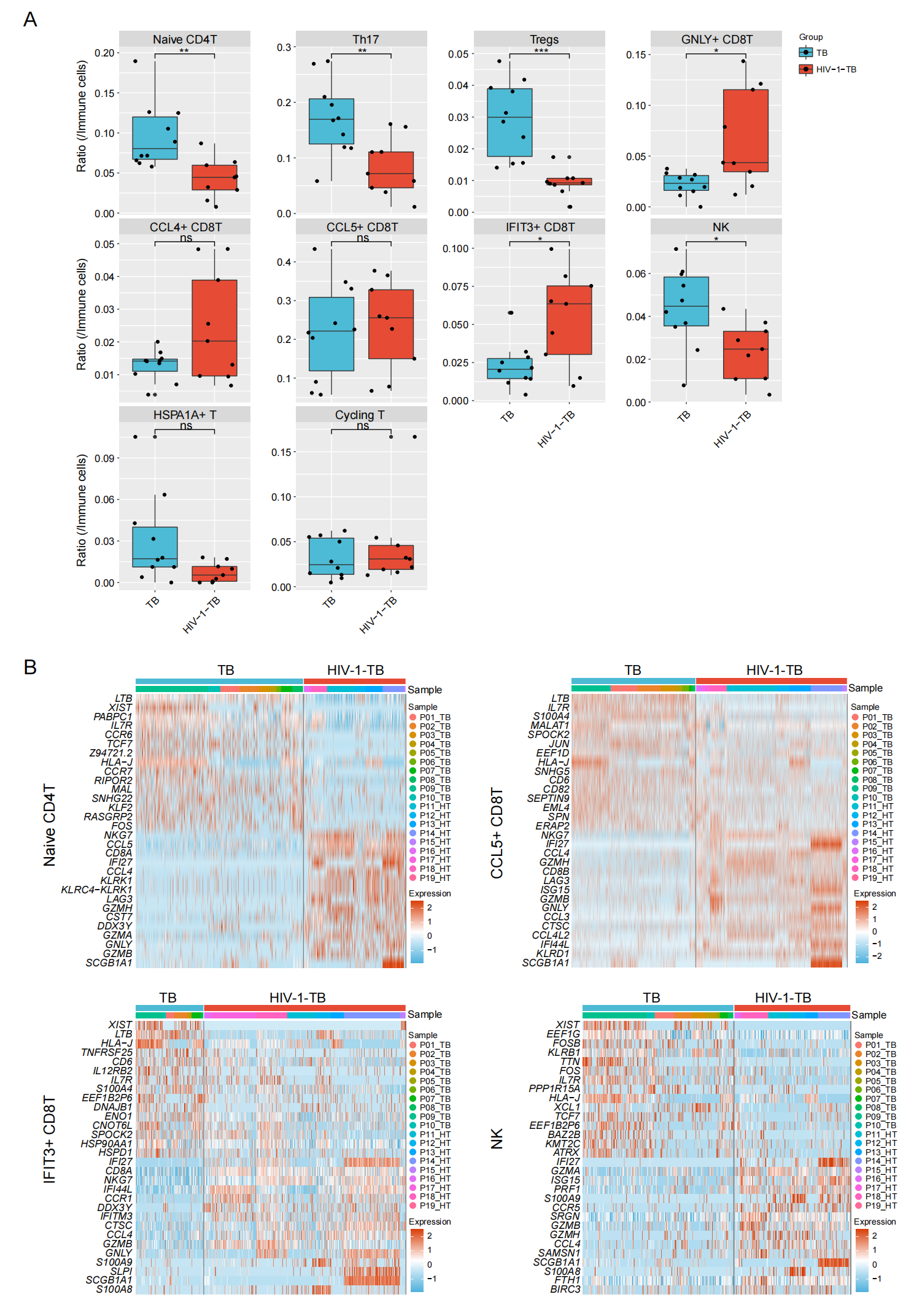

Supplement: jiae042_Supplementary_Data [file jiae042_supplementary_data.zip › FigureS5.tif]
